# Navigating the Healthcare System With Chronic Illness: A Qualitative Study of Caregiver Experiences

**DOI:** 10.1111/scs.70094

**Published:** 2025-08-07

**Authors:** Malene Missel, Geana Paula Kurita, Merlin Kirstine Egeberg Lindblad, Louise Laursen, Inge Eidemak, Ditte Tang Johansen, Sille Larsen, Nanna Witting, Karin Piil, Linda Kahr Andersen

**Affiliations:** ^1^ Department of Cardiothoracic Surgery Copenhagen University Hospital Copenhagen Denmark; ^2^ Department of Clinical Medicine University of Copenhagen Copenhagen Denmark; ^3^ Section of Palliative Medicine, Department of Oncology and Multidisciplinary Pain Centre, Department of Anaesthesiology, Pain, and Respiratory Support Rigshospitalet – Copenhagen University Hospital Copenhagen Denmark; ^4^ Section of Palliative Medicine, Department of Oncology Rigshospitalet, Copenhagen University Hospital Copenhagen Denmark; ^5^ Copenhagen Neuromuscular Center, Department of Neurology Rigshospitalet, Copenhagen University Hospital Copenhagen Denmark; ^6^ Department of Oncology Copenhagen University Hospital Copenhagen Denmark

**Keywords:** chronic illness, healthcare professionals, informal caregivers, patient‐centred care, phenomenological hermeneutics

## Abstract

**Background:**

Chronic illness management often requires ongoing self‐care by patients, along with complex interactions with healthcare professionals. Informal caregivers play a crucial role in supporting individuals with chronic conditions; however, they often feel unsupported and overlooked within the healthcare system. Understanding the challenges caregivers face when interacting with healthcare professionals is essential for improving caregiver support and patient outcomes.

**Aims:**

This study aims to generate new knowledge about how informal caregivers experience their role and navigate interactions with healthcare professionals in the context of chronic illness care.

**Methods:**

We used a phenomenological hermeneutical approach inspired by Ricoeur's philosophy. Data were collected through individual interviews with eleven diverse caregivers of persons with chronic illnesses, focusing on their interactions with healthcare professionals. The interviews were analysed using interpretive methods to uncover deeper meanings in the caregivers' narratives.

**Findings:**

Four main themes emerged, highlighting caregivers' interactions with healthcare professionals: Trust and tension in specialised care, Recognising the individuals behind the caregiver roles, The caregiver as translator and entrepreneur between patient and healthcare professionals, and The silent struggle of caregivers. Caregivers expressed trust and security in specialised hospital settings, but also frustration over inconsistent communication and lack of proactive support. They acted as essential mediators and entrepreneurs in the healthcare process, facing significant burdens. Caregivers also emphasised the need for more personalised attention and recognition from healthcare professionals.

**Conclusions:**

Caregivers often serve as translators, advocates, and coordinators, underscoring the need for healthcare professionals to better integrate and support them. To improve clinical care, healthcare systems must adopt more inclusive practices, enhance communication, and value caregivers' expertise, guided by frameworks such as family systems theory and Habermas' system and lifeworld theory.

## 1 Background

Optimal management of chronic health conditions involves multifaceted daily self‐management routines for patients and often complex interactions with healthcare professionals (HCP) [1]. The involvement of informal caregivers such as spouses, adult children, relatives, friends, or neighbours in the patient care provides a significant source of day‐to‐day disease management, and many adults with chronic illness depend on such caregiver support [2, 3]. Informal caregivers are usually unpaid non‐HCP who provide for the health and well‐being of the person with chronic illness [4]. The caregiver support dynamics vary according to the patient's functional status and required level of care. Informal caregivers of individuals with severe functional constraints frequently engage directly in practical health‐related tasks on behalf of the person living with illness. Conversely, informal caregivers of chronically ill but functionally independent adults generally may aid in facilitating the patient's own self‐management [5]. This can be in the form of daily decisions regarding treatment management, coordinating healthcare efforts across various providers, and fostering behavioural lifestyle changes, such as enhancing diet or encouraging self‐monitoring [1].

Worldwide, chronic diseases such as cardiovascular diseases, diabetes, cancer, and chronic respiratory diseases account for approximately 74% of all deaths [6]. In Denmark, around every third adult lives with at least one chronic condition, with the most common being diabetes, heart disease, arthritis, COPD, and cancer [7, 8, 9]. The increasing prevalence of chronic illness underscores the need to understand the roles and challenges faced by those supporting individuals in managing these conditions.

It is furthermore reported how living with others and having a caregiver are factors associated with improved health‐related quality of life and increased engagement in self‐care activities for individuals living with chronic illness [10]. Support from caregivers has great potential to help people with chronic illnesses better manage their conditions [2, 11]. However, being a caregiver of and caring for a person with a chronic illness can be a burden and negatively influence the caregiver's everyday life, quality of life, health, and overall well‐being [12, 13, 14, 15]. Due to the ongoing nature of chronic illnesses, it is not uncommon for carers to spend many years undertaking care tasks, such as navigating health services. Despite their integral role, caregivers frequently report feeling unsupported and overlooked by the healthcare system [16, 17], and they are described as a “hidden patient group,” “hidden carers” or “outsiders” in need of greater recognition and support from HCP [12, 18, 19]. Caregivers often point to a lack of structured involvement in care planning, limited access to tailored information, and insufficient guidance on navigating the healthcare system as key areas where HCP fall short [20, 21, 22, 23]. They also report inadequate emotional and psychological support, especially in long‐term caregiving situations. Proactive communication, systematic inclusion in decision‐making processes, and practical training in symptom management are forms of support that are often unavailable but could significantly reduce caregiver burden.

Recognising these challenges underscores the importance of exploring caregivers' experiences when interfacing with HCP. Understanding how caregivers navigate and interact within the healthcare system is essential for improving their support and ultimately enhancing patient care outcomes [24]. While existing literature acknowledges the essential role of caregivers, there remains a gap in understanding the nuanced experiences of caregivers as they interact with HCP [3, 25]. Existing research primarily focuses on the practical aspects of caregiving tasks and their impact on caregivers' lives, yet there is limited exploration into how caregivers perceive and navigate their roles within the healthcare system. Therefore, this study aims to investigate and gain insight into the experiences of informal caregivers of individuals with chronic illnesses when engaging with HCP.

## 2 Method

This study explores caregivers' lived experiences through a phenomenological hermeneutical approach inspired by Ricoeur [26, 27]. Phenomenology serves as the epistemological foundation for understanding pre‐reflexive lifeworld experiences, while hermeneutics uncovers deeper meanings in their engagement with HCP through language [26, 28].

### 2.1 Setting and Study Participants

The participants in this study were caregivers of individuals undergoing treatment for chronic diseases. Specifically, we focused on caregivers of patients with kidney failure treated by haemodialysis, cystic fibrosis, intestinal failure treated by parenteral nutrition, or patients with generalised myasthenia gravis. These conditions require lifelong treatment, frequent hospital check‐ups, significant symptom management, and ongoing self‐care [29]. The caregivers included in the study were identified by the patients as key individuals involved in their care. Participant information is presented in Table 1.

**TABLE 1:** Participant information.

Participants	*n* = 11
Age, years (range)	25–72
Gender (female/male)	8/3
Time since diagnose (years)	4–46
Relationship to the ill person	
Partner	9
Sibling	1
Child	1
Disease of the ill person	
Kidney failure	4
Cystic fibrosis	2
Intestinal failure	3
Myasthenia gravis	2
Education	
Primary school or less	4
Further education	6
Completed secondary school or higher	1
Job situation	
Working full‐time	5
Working part‐time	4
Early retirement	1
Retired	1
Family situation	
Children living at home	*n* = 6

A purposive sampling method was used to ensure a diverse group of participants [30]. Eleven caregivers of individuals with different chronic illnesses, varying relationships with the patient, and a range of ages, genders, and geographic locations across Denmark were included. This diversity was intended to capture a wide array of experiences related to living with a chronically ill person and engaging with HCP, aligning with the study objective. In assessing the adequacy of sample size, the concept of “information power” guided our approach [31]. Information power posits that as the relevance and richness of information within the sample increase, fewer participants are required. Through purposeful sampling and inclusion of a diverse participant group, the sample size was deemed sufficient to comprehensively address the research aims.

In keeping with phenomenological hermeneutic methodology [26, 28, 32, 33, 34], no formal inclusion or exclusion criteria were applied beyond the requirement that participants were informal caregivers to individuals receiving long‐term, specialised treatment for chronic illness. The aim was not to recruit a representative sample, but rather to ensure variation in lived experiences across different illness trajectories, caregiver roles, and life situations. This openness aligns with the epistemological commitment of phenomenology to explore the phenomenon as it appears to diverse individuals in their everyday lives. All participants were caregivers of patients receiving specialised treatment at university hospital departments. The recruitment process involved a project nurse (SL) and a researcher (LKA) who informed potential participants about the study's purpose. Caregivers who agreed to participate were scheduled for an interview. All interviews were coordinated in collaboration with the caregivers, who selected a suitable time in dialogue with the research team. All interviews except one were conducted face‐to‐face in a quiet and comfortable room at the hospital, designed to support confidential conversations. One interview was conducted by telephone at the participant's request. These interviews were carried out by an experienced clinical psychologist (DTJ), a senior palliative consultant (IE), and a research physiotherapist (LKA). The interviewers ensured that they did not engage in clinical consultations with the patients or caregivers involved in the study.

### 2.2 Data Collection

Data for this study were gathered through individual interviews [26]. Caregivers were encouraged to narrate about matters they deemed significant, with follow‐up questions posed from a dialogical standpoint to deepen the exploration. To maintain alignment with the purpose of the study, a set of open‐ended questions (listed in Table 2) was prepared. These questions served as prompts to guide the dialogue towards specific aspects related to the caregivers' engagement with HCP. However, the primary focus remained on allowing the caregivers to lead the conversation and articulate their perspectives. Each interview lasted, on average, 50 min and was audio‐recorded to capture nuances in the caregivers' expressions. Subsequently, the recordings were transcribed verbatim to preserve the authenticity and richness of the data.

**TABLE 2:** Interview questions for caregivers of persons with a chronic illness.

Can you talk about your experiences of being a caregiver or living with a person affected by chronic illness? In relation to your personal experience of illness and treatment, can you talk about how your experiences of meeting the healthcare system and healthcare professionals are like? Do you recall any situations in which you experienced something related to meeting the healthcare system and healthcare professionals that interfered in your ways of caregiving? What is important for you to maintain a meaningful collaboration with healthcare professionals?

### 2.3 Data Analysis

Interpretation serves as the central methodology in phenomenological hermeneutical work, as outlined by Ricoeur [26, 28]. Ricoeur underscores that the written text, exemplified by the transcribed interviews in this study, is not a mere extension of spoken words; rather, he posits that language undergoes a transformation when transcribed. When converting spoken words into written form, the description of lived experiences becomes detached from the event, liberating the meaning of the text from the narrators' underlying intentions. This liberation enables researchers to interpret and unravel the issues indicated by the text. Language, including texts, harbours connotations that can only be accessed through an interpretive process, according to Ricoeur. This interpretive process, he argues, is an endless spiral encompassing three levels: a naive exploration of the overarching meaning the text conveys, a linguistically oriented structural analysis, and an in‐depth comprehensive understanding [28].

The initial naive interpretation involves a superficial engagement, entailing the reading and re‐reading of transcriptions to capture an initial understanding. The structural analysis provides insights into the text's organisation, extracting words and sentences that indicate recurring issues and themes throughout. The comprehensive understanding aims to grasp the meaning and breadth of the issues and themes in the text, offering a more profound and sophisticated comprehension of the lifeworld phenomena evident in the caregivers' descriptions. Experienced qualitative researchers and clinicians (MKEL, LL, DTJ, LKA, and MM) conducted the data analysis, engaging in collaborative discussions. Data and interpretations were reflected upon and discussed at all levels in the analytical and interpretive process before being reviewed with the wider research team, which encompassed educational backgrounds and clinical experiences in medicine, nursing, physiotherapy, psychology, and social work. The four main themes presented in the findings emerged through this iterative interpretive process. In line with the Ricoeur‐inspired approach, we did not develop formal sub‐themes. Rather, the themes reflect synthesised and interpretive understandings of recurring patterns and essential meanings identified across the narratives.

## 3 Findings

Four themes presented in this section express the meaning of how it is experienced to engage with HCP as a caregiver of patients receiving treatment for a chronic illness. For an overview of these themes and their interpretive content, see Table 3.

**TABLE 3:** Findings.

Main theme	Interpretive description
Trust and tension in specialised care	Caregivers expressed trust in specialised hospital care but also frustration over lack of follow‐up and systemic fragmentation. Trust coexisted with responsibility and emotional burden
Recognising the individuals behind the caregiver roles	Caregivers described the need for personalised interaction with HCP and felt frustrated when treated as anonymous or overlooked. They emphasised the importance of clear, empathetic communication
The translator and entrepreneur between patient and HCP	Caregivers often acted as intermediaries, advocates, and organisers of care. They described taking on extensive responsibilities and lacking adequate support from the healthcare system
The silent struggle of caregivers	Caregivers reported emotional strain, invisibility, and lack of recognition from HCP. They expressed a need for acknowledgement of their role and well‐being

### 3.1 Trust and Tension in Specialised Care

The caregivers of individuals with chronic diseases in this study express a sense of security and trust in the expertise and knowledge at the specialised university hospitals concerning the treatment of their disease. They repeatedly mention these settings as places where they and the patients felt recognised and well cared for. However, this trust is not without its limits. Despite their overall positive experiences, caregivers also shared frustrations related to fragmented communication, lack of follow‐up, and feeling responsible for ensuring adequate care when support systems failed. This ambivalence reveals how trust can coexist with pressure and burden in long‐term caregiving:

…before she [the patient] came under the wings of Rigshospitalet [Danish National University Hospital]…, it wasn't easy. It was good that she did, because she is much more comfortable with it, we both are, than having to go to the, with all due respect, slightly smaller hospitals…, you can't expect them to know anything about myasthenia. (J)

Caregivers express gratitude for the special attention they and the patient receive, feeling seen and heard as an integral part of the treatment process as expressed by this caregiver: “The fibrosis department has been amazing, and we are both very grateful for that. Everyone has been attentive and responsive to our needs” (E). Despite these positive experiences, caregivers can also experience frustration over a lack of information about treatment options and rehabilitation, as well as a lack of proactivity across the healthcare system. They mention feeling compelled to take the initiative themselves to ensure the patient's treatment, and feeling abandoned and overlooked by the HCP:

P [the patient] was also finally referred for rehabilitation, but it was only when I put my foot down and said, now we must be referred, end of story! Because we didn't get any information from the hospital that it was possible to be referred to rehabilitation… the last seven months maybe, we haven't seen or heard anything from them [the hospital/HCP] at all. I feel like we've been completely forgotten. (K)

These contrasting experiences suggest that while caregivers value being recognised and supported by HCP, they also face a lack of organisational responsiveness that undermines their resilience. When caregivers encounter proactive, attentive care, it strengthens their motivation and sense of shared responsibility. Conversely, when support structures fail, caregivers feel abandoned and burdened with navigating a fragmented system alone. This highlights a need for consistent, empathetic communication and better organisational support that empowers caregivers rather than exhausts them.

Even with these challenges, the analysis shows that caregivers actively participate in the treatment process and contribute to constructive collaboration between patient, caregiver, and HCP as expressed by this caregiver: “It's a big common project and interest for all of us involved, that's what I call it. I pretty much come along to everything when there are appointments at the hospital because you can't remove myasthenia” (J). Caregivers' dedication and cooperation build trust and security, emphasising the importance of a collective approach to treatment. However, their continuous involvement in appointments can also be burdensome, as they often feel obligated to attend due to the chronic nature of the disease, highlighting the challenges they face in balancing support with personal and practical demands.

Furthermore, caregivers feel a significant responsibility for the patient's well‐being and treatment, especially when local hospitals lack the necessary expertise. This responsibility places a heavy burden on caregivers, who feel compelled to act as the patient's advocates to ensure proper treatment:

…particularly when we are at the local hospitals where they don't have the expertise and don't know much about the disease, I feel it becomes my responsibility to explain to the healthcare professionals. Several times, I've had to push and be persistent to get my husband transferred to Rigshospitalet [Danish National University Hospital], where they are familiar with his intestinal disease, and it's a bit tough to be the one who must speak up…. (B)

This perspective highlights the need for possible improvements in local hospitals' knowledge and competencies to manage complex chronic conditions, as well as enhanced communication between HCP and patients/caregivers to ensure more effective treatment and positive experiences.

### 3.2 Recognising the Individuals Behind the Caregiver Roles

Caregivers express a desire for more individualised care, tailored to both the patient's and caregiver's personality and needs. The analysis highlights the importance of a varied approach from HCP in their interactions with patients and caregivers. Discrepancies in HCP engagement significantly affect the experience, security, and trust in treatment.

I would have liked them to have been better at differentiating between patients and understanding who Y [patient] and I are, and how we approach this. There were differences among the nurses in how much they engaged with us; some were very present and attentive, while with others, it felt like they were just waiting to get back to what mattered to them. Imagine if there was a big sign in the nursing and doctor's office saying: Every dialysis patient is different! It's about who you are as individuals…. (A)

Despite their vulnerable and emotional situation, caregivers may feel unnoticed by HCP, leading to feelings of frustration and isolation. On the other hand, caregivers also describe positive experiences of HCP as authority figures who simultaneously foster collaboration and involvement of the patient and family in decision‐making processes. This underscores the importance of recognising the caregiver's role as an essential part of the patient's support system. Caregivers also express a desire for HCP to convey information in an understandable manner, enabling caregivers to actively participate in decision‐making processes and understand the patient's treatment as expressed by this caregiver: “P [patient] and I are not very good at understanding the information, and I must go home and think about what it means… Sometimes it's a bit difficult to understand your language, as if I can't get it right in my head…” (K).

Caregivers also describe experiences of alienation and discomfort in the healthcare system. They perceive the system as primarily oriented towards HCP and insufficiently tailored to the needs of patients or their caregivers. This perception creates feelings of powerlessness and frustration as caregivers feel overlooked and marginalised in their encounters within the healthcare system. Moreover, there can be a communication gap between HCP and caregivers. HCP use of technical and incomprehensible language, along with their tendency to avoid specific questions and concerns, creates barriers to meaningful dialogue. This is exemplified in this excerpt: “They [HCP] talk about percentages and statistics, and typically they also say something like, well, we can't say anything about that…” (H). The difference in perspective between HCP and caregivers are thus illuminated, and in extension, caregivers describe how there can be a tendency to primarily focus on treating physical symptoms in patient care and treatment. Caregivers may experience insufficient support or guidance from HCP regarding non‐physical issues. This creates feelings of frustration for the caregivers.

These experiences demonstrate that caregivers' sense of inclusion and empowerment is closely tied to how empathetically and individually HCP interact with them. When caregivers feel acknowledged, informed, and understood, they are more capable of contributing actively and constructively to the care process. In contrast, when caregivers encounter indifference or incomprehensible communication, it reduces their ability to act, weakens trust, and may diminish their motivation. Strengthening empathetic, personalised, and accessible interaction is thus essential to support caregivers' resilience and engagement.

### 3.3 The Translator and Entrepreneur Between Patient and HCP

The analysis illuminates the significant role that caregivers undertake as translators and mediators between the patient and HCP. Beyond literal translation, caregivers navigate emotional reactions and convey nuanced needs, shouldering the responsibility of articulating the patient's concerns in a comprehensible manner. Moreover, caregivers serve as conveyors of health information to the patient, which can be crucial for the patient's understanding of the situation.

Caregivers describe themselves as entrepreneurs who must ensure the proper treatment and care of the patient, while facing challenges such as a lack of communication from HCP. This entrepreneurial role involves a proactive approach to organising and coordinating the patient's care, which can be challenging: “The tough part has been the fact that I can never just stay away… I'm like the entrepreneur all the time, making sure… yeah, like making sure he gets the penicillin he's on, etc.” (B). Additionally, caregivers take on roles such as the patient's advocate, nurse, and even doctor, where they feel responsible for ensuring the patient's interests, performing nursing tasks, and making treatment decisions, as expressed by this caregiver: “I have to be my wife's advocate, I have to be her nurse, I have to be her doctor. And sometimes I have to tell the doctor: do what we know helps the best” (F). This shows the extensive responsibility that caregivers undertake in caring for the ill person. The analysis also shows how caregivers experience being assigned additional responsibilities by the hospital, which can increase their burden.

Despite their crucial role, caregivers may experience isolation in managing the ill person's care without adequate guidance or support from HCP. This perceived lack of support can lead to frustration and feelings of inadequacy. Moreover, caregivers express insufficient information from hospitals about the specific chronic illness affecting the ill person, leaving them to seek answers independently. External support from patient organisations and networks such as Facebook becomes crucial in providing caregivers with the necessary information and support. However, this information about the disease is not always enough, and the caregivers express feelings of anxiety and confusion about the patient's illness and its trajectory:

It was my sister‐in‐law who said that there was a Facebook page. And through that, we were simply told that there was also something called Muskelsvindfonden [Danish patient organization], and they could help by telling us what it [the disease] was. Because we got no information about it otherwise, from the hospital. And only then did I begin to understand what was wrong with him [the patient]. But it took some time for me to calm down when I was told that you can die from it, but that it's not something that normally happens. (K)

In navigating these challenges, caregivers adopt an entrepreneurial mindset, proactively seeking resources and advocating for the ill person's needs within the healthcare system. They take on roles beyond traditional caregiving, including “researcher” and advocate, ensuring comprehensive care and informed decision‐making.

Caregivers may feel ignored or unheard by hospital staff when trying to communicate their needs and wishes for treatment to the many different HCP they encounter. This feeling is reinforced by the lack of knowledge about their family's specific situation and medical history among HCP, resulting in repeated explanations and a feeling of not being taken seriously. Caregivers express how they often have a deeper understanding of the patient's body and disease and expect that their contributions should, therefore, be considered in healthcare decisions:

We've been through this journey for years now, and I know my husband's symptoms and reactions better than anyone. It's disheartening when I have to explain everything anew to every new doctor or nurse we encounter. I expect our experiences and input to be taken seriously. (G)

These findings underscore how caregivers, in the absence of sufficient support from the healthcare system, are compelled to adopt complex roles that demand both emotional labour and organisational skills. While many caregivers take on these responsibilities with commitment and resourcefulness, the lack of formal recognition and structured support can lead to frustration, emotional exhaustion, and a sense of marginalisation.

### 3.4 The Silent Struggle of Caregivers

The results of the analysis indicate that despite their deep understanding of the patient's condition and experiences, the caregivers express a lack of recognition and understanding from HCP. The unique knowledge that caregivers possess because of their close relationship with the patient is not always appreciated or listened to by HCP, creating frustration and a sense of not being understood:

…and there were three consultants and nurses saying everything was fantastic. In the end, I had to shout that nothing here is fantastic! If barely getting through life, having no energy, misplaced anger, and being unable to eat is ‘great,’ then fine – but not by my standards! The look I got from the doctors could kill; I felt like the biggest witch in the world. (B)

Furthermore, caregivers describe how HCP do not often show specific interest in their well‐being and emotional state. This can result in caregivers feeling like shadows or overlooked figures in the patient's treatment process. This lack of attention to caregivers' well‐being worsens the feeling of not being seen or heard as individual persons but rather as secondary players in the patient's disease trajectory: “The caregivers become shadows. There's the disease and the healthcare system, and we as caregivers in the middle must make sure that the patient has a good life and a tolerable life, but this means that the caregivers are lost in a way…, they are not seen at all” (I).

Moreover, caregivers point out a tendency to be confined to stereotypical roles, especially if the focus is only on patients with specific types of diseases such as cancer, as expressed by this caregiver: “When you walk around Rigshospitalet [Danish National University Hospital], there are huge posters saying that: “Behind every cancer patient there is a caregiver”. I thought about burning them, seriously. I said to my wife every time, there's a caregiver behind every other patient too…” (F). This further reinforces the feeling of not being recognised in the healthcare system and underscores the need for a more inclusive approach from HCP. They seek opportunities for conversations and support that can address their specific needs as caregivers, and they long for the feeling of being seen, asked, and heard:

I wish the doctors and nurses would also sometimes ask how you're doing as a caregiver, and not just focus on the patient even though he is the most important because he is the one having the disease. But I also have my own struggles and emotions in this journey, and sometimes it feels like no one sees that…. (B)

Thus, this theme encapsulates the often‐overlooked emotional and psychological burden that caregivers bear while supporting the person with chronic illness. While the patient rightfully commands attention due to their illness, caregivers also experience profound challenges that impact their daily lives. The dual nature of the caregiver role is underscored as they not only provide physical and emotional support to the patient but also navigate their own journey of coping with uncertainties and emotional challenges. This plea reflects a deeper need for recognition and validation from HCP, emphasising that caregivers are not just bystanders in the patient's healthcare journey but active participants whose well‐being is intertwined with that of the patient. The absence of emotional support and recognition from HCP not only contributes to caregivers' sense of invisibility but also threatens their long‐term capacity to sustain the caregiving role. Empathetic encounters—where caregivers are seen, heard, and asked about their own well‐being—could provide much‐needed validation and strengthen their resilience.

## 4 Discussion

Our study underscores the significant challenges that caregivers of chronically ill persons encounter when navigating the healthcare system. Despite often possessing profound knowledge about the patient's condition, caregivers can perceive their insights as overlooked by HCP, thereby assuming responsibility for ensuring appropriate care and treatment. Consequently, HCP risk overlooking key aspects related to the patient's disease and management.

The findings show how caregivers trust specialised university hospitals for their expertise but are frustrated by the lack of proactive communication, information, and being acknowledged for the important role they fulfil. Inconsistent engagement exacerbates these frustrations, contrasting with positive experiences that emphasise the importance of including caregivers in decision‐making processes. The professional focus of the healthcare system sometimes marginalises caregiver needs, resulting in communication gaps and a sense of powerlessness. Previous research highlights the dyadic nature of patient‐caregiver relationships in managing chronic illness, where both individuals mutually influence each other and adjust to the challenges posed by the illness [35, 36, 37]. From a clinical standpoint, it is imperative for HCP to recognise the patient‐caregiver dyad as the unit of care. Understanding the shared illness appraisal and collaborative disease management within this dyad is critical for tailoring interventions directed to both the patient and caregiver to improve patient self‐care management [35].

Despite an increasing interest in integrating caregivers into healthcare settings, practical interventions for effectively assessing caregivers' needs remain limited, and there is significant variability in current approaches [25]. A key consideration when engaging with caregivers lies in navigating the tensions between individual autonomy and the shift towards family‐centred care, where caregivers play essential roles as partners in the care process [3]. Emerging frameworks such as family systems theory hold promise in assessing family function and supporting the entire family [38, 39, 40]. Within this theory, families function as complex systems, where members interact with and influence each other. The theory posits that any change in one member affects the whole system, which is highly relevant in chronic illness trajectories where caregiving and emotional burden are closely linked. Applying this theory helps HCP view caregivers not merely as supporters but as co‐affected individuals whose needs and experiences directly influence the patient's care and outcomes. The theory helps HCP understand the family as a dynamic unit and recognise the interconnectedness of individual family members' experiences with health and illness.

The challenges outlined in our findings may therefore emphasise the critical need for a more family‐focused approach, recognising caregivers as a vital support system for patients. Family‐focused approaches, such as family conversations, have demonstrated significant benefits across various chronic illnesses, including improved well‐being, enhanced perceived control over the illness, and strengthened family cohesion [41, 42, 43]. These findings highlight the importance of integrating family‐centred care to address the needs of both patients and their families in a person‐centred manner. This approach could be adapted and tested within this population, potentially offering similar benefits. By viewing caregivers as part of a larger family system, HCP can gain insights into caregivers' experiences and challenges, thereby enhancing support strategies. However, research also highlights the need to be sensitive to both the caregiver's and the cared‐for person's perceptions of their family roles to improve the uptake of support [18].

As translators and entrepreneurs, caregivers in our study play a crucial role in ensuring treatment continuity, yet they often feel overwhelmed and unsupported. Despite their invaluable insights, caregivers frequently perceive that their contributions are overlooked, underscoring the need for better recognition and inclusion in the care process. Other studies have also reported caregivers acting as patient advocates, demonstrating their engagement and preparedness on behalf of patients [44, 45]. These findings underscore the imperative for HCP to adopt a more inclusive approach, where caregivers are acknowledged and integrated into the care process to enhance outcomes for both patients and caregivers. Habermas' system and lifeworld theory [46] provides a pertinent lens through which to understand many of the challenges faced by caregivers. According to Habermas, the healthcare system is governed by “system rationality”—a logic oriented towards efficiency, standardisation, and control—while the lifeworld represents the domain of personal meaning, relationships, and lived experience. In our study, caregivers operate within their lifeworld, where caregiving is not merely a task, but an existential, emotional, and moral commitment shaped by love, responsibility, and accumulated knowledge about the patient. When these lifeworld experiences are not recognised by the system, a tension arises and caregivers feel alienated, dismissed, and burdened. Understanding this clash helps explain why caregivers experience frustration and emotional strain when their personal, situated knowledge is disregarded in clinical encounters. This prioritisation can lead to caregivers feeling marginalised and their insights undervalued. In contrast, caregivers operate within their lifeworld, where understanding of the patient's condition and their caregiving role is deeply intertwined with personal experiences, cultural contexts, and emotional bonds. When their lifeworld experiences are not acknowledged within the healthcare system, it can create disconnection and feelings of frustration. The unseen cost of informal care has been revealed as an expensive and temporal burden, illustrating common circumstances for people living with chronic illness [3]. This suggests that healthcare systems need to develop integrated care solutions that address both the healthcare system and the lifeworld, aiming to alleviate the burden on caregivers. As is well‐known, individuals with chronic health conditions often rely heavily on their caregivers for much of their necessary care and daily support. Society would encounter substantial challenges in meeting these needs without their contribution [47]. Therefore, it is crucial for HCP to recognise and collaborate with these caregivers to enhance the quality of life for the ill person [19].

### 4.1 Limitations

This study offers valuable insights into caregivers' experiences within the healthcare system, yet it has limitations. The study did not fully explore factors such as the impact of familial relationships (e.g., siblings, spouses/partners, parents, children, friends) and emotional dynamics (e.g., closeness, dependency, difficulty) due to a limited sample. Additionally, variables like gender, social class, network size, and life phase of caregivers were not thoroughly investigated. However, such factors can significantly influence caregiving experiences [48]. In this study, participants' shared experiences are collectively presented, focusing on commonalities among the caregivers. This approach amplifies their shared voices. However, it also results in certain individual phenomena not being fully described. Thus, there is selectivity in representing voices, prioritising prevalent and crucial findings from the interviews. To validate these selections empirically, five researchers have read and re‐read the interviews alongside the drafts on interpretation, ensuring robustness in the study's conclusions [30].

The study sample comprised eight female and three male caregivers. It is well‐documented that female caregivers often perceive caregiving as an inherent responsibility, a natural part of life. Moreover, research indicates that female caregivers generally report lower life satisfaction compared to their male counterparts [19]. These gender‐related factors should be considered when interpreting the transferability of the study findings. Not all caregivers are equally involved or willing to identify as such, highlighting the complexity of caregiver roles [35]. This understanding is crucial for appreciating the varied dynamics and challenges faced by caregivers. Support strategies actively engaging caregivers are imperative through family‐focused interventions. While our study advocates for HCP to engage caregivers, involvement levels vary, requiring a nuanced approach. Though some caregivers are reluctant to define themselves as such and resist adopting the label [18], this phenomenon was not observed in our study but should be considered in broader applications.

## 5 Conclusion

This study highlights the crucial role of caregivers in ensuring quality care for individuals with chronic illnesses, despite challenges in the healthcare system. Caregivers trust specialised hospitals for expertise but express frustration over inconsistent communication and lack of recognition. Acting as translators, entrepreneurs, and advocates, they provide essential insights for patient‐centred care. To better support caregivers, healthcare systems must adopt inclusive, family‐focused practices, enhance communication, and integrate caregivers into decision‐making. Family systems theory and Habermas' system and lifeworld theory offer valuable perspectives on these challenges.

## Author Contributions

I.E., L.L., S.L., M.K.E.L., and D.T.J. conceptualised, designed, and obtained funding for the research. I.E., L.L., S.L., M.K.E.L., L.K.A., and M.M. were involved in protocol development, gaining approval, and patient recruitment. D.T.J., I.E., and L.K.A. completed data collection. L.L., M.K.E.L., L.K.A., and M.M. completed data analysis and wrote the first draft of the manuscript. All authors reviewed and edited the manuscript and approved the final version.

## Ethics Statement

The study received approval from the Danish Data Protection Agency under the Capital Region of Denmark (journal number: P‐2020‐111) and adhered to the guidelines set forth by the Danish Ethics Research Committee.

## Consent

The caregivers involved in the study were provided with comprehensive information both verbally and in writing. They were assured that their data would be handled with utmost confidentiality, and any information that could be traced back to them or the individuals under their care would be pseudo‐anonymised. Participants were explicitly informed of their right to withdraw from the study at any point without impacting their ongoing treatment. Written consent was obtained from all participants.

## Conflicts of Interest

The authors declare no conflicts of interest.

## Supporting information


**File S1:** scs70094‐sup‐0001‐FileS1.pdf.

## Data Availability

The first author/corresponding author (MM) and the last author (LKA) have full control of all primary raw data (interview transcripts) and allow the journal to review our data if requested. The data generated during and/or analysed during the current study are available from the first author on reasonable request. All raw data are written in Danish. Data are stored in a locked file cabinet in a locked room at the Copenhagen University Hospital as requested by the Danish Data Protection Agency.
